# Public Awareness regarding the Differences between Ophthalmologists and Optometrists among Saudi Adults Living in Riyadh: A Quantitative Study

**DOI:** 10.1155/2018/7418269

**Published:** 2018-09-30

**Authors:** Mohammed Hamad Aldebasi, Nasser Abdalazez Alsobaie, Abdulrahman Yousef Aldayel, Khalid Mousa Alwusaidi, Tariq Alasbali

**Affiliations:** ^1^College of Medicine, Al Imam Mohammad Ibn Saud Islamic University (IMSIU), Riyadh, Saudi Arabia; ^2^Ophthalmology Department, College of Medicine, Al Imam Mohammad Ibn Saud Islamic University (IMSIU), Riyadh, Saudi Arabia

## Abstract

Awareness of the patients to the differences between ophthalmologists and optometrists has been recognized as an important factor influencing patient utilization of available eye care services. This study aimed at assessing the public's knowledge of the differences between ophthalmologists and optometrists among the residents of Riyadh, Saudi Arabia. An online questionnaire was administered to adults in Riyadh, Saudi Arabia, from January to February in 2018. The number of the respondents was 1579. Results show that 50% (*n*=789) of the participants had fair knowledge of the differences between ophthalmologists and optometrists, while 32% (*n*=505) had poor knowledge. After multivariate logistic regression analysis, there was a significant association between satisfactory knowledge and visiting an optometrist (odds ratio (OR): 0.75, 95% CI: 0.57–0.98), age older than 26 (OR: 1.73, 95% CI: 1.17–1.19), higher level of education (OR: 1.92, 95% CI: 1.54–2.4), and wearing spectacles (OR: 0.57, 95% CI: 0.45–0.73). Given the low level of public knowledge regarding the differences in the duties between ophthalmologist and optometrists among a Saudi population, there is great potential for general population education through awareness campaign and social media regarding eye care provider's duties and practices.

## 1. Introduction

Both the World Health Organization (WHO) and the International Agency for the Prevention of Blindness (IAPB) agreed in their joint framework of VISION 2020 [1] that appropriate access to eye care professionals is needed to foster vision health worldwide. The eye care services including their curative, preventive, and promotive types emphasize the importance of the knowledge in how to choose the qualified practitioner who has the appropriate training and skills. Eye care, in general, is provided mainly by ophthalmologists and optometrists; regardless of the differences in the training and license requirements, their roles in providing eye care services are overlapped [2]. There are numerous key differences between ophthalmology and optometry. For instance, the length of training, in ophthalmology, the specialist must first complete the medical school and finish 4 years of medical training in the ophthalmology department in a hospital (total of at least 11-12 years depending on the country) [3]. Moreover, ophthalmologists specialize in a specific area of surgical or medical eye care, and this is called a subspecialty. This subspecialist usually completes one or two years of extra, more comprehensive training known as the fellowship program in one of the subspecialty areas such as retina, glaucoma, cornea, neurology, pediatrics, and plastic surgery, along with others more [4]. However, to be optometry specialist, you must graduate the optometry school and finish the related internship (total of at least 4-5 years depending on the country). For duties, an ophthalmologist has the authority to diagnose and treat all eye diseases, perform eye surgeries, prescribe medications, and fit eyeglasses/contact lenses to correct vision problems. Many ophthalmologists are also involved in scientific research on the eye care and vision diseases. On the other hand, an optometrist is primarily involved in performing eye exams and vision tests, prescribing and dispensing corrective lenses, detecting certain eye abnormalities, and prescribing some medications for certain eye diseases [3]. There is mutual beneficial relationship in that ophthalmologists may refer patients to optometrists for optical aids and vision therapy, particularly, contact lens fittings or low-vision rehabilitation. Additionally, optometrists may refer patients to ophthalmologist for further assessment, treatment, and surgical management of ocular diseases. Both optometrists and ophthalmologists can prescribe certain medication for eye health and perform surgeries [5]. However, this defers depending upon local regulations. In Saudi Arabia, ophthalmologists are usually responsible of prescribing a wider range of medications as well as performing all forms of surgery, while optometrists are not given the responsibilities of physicians; they can refer a patient to ophthalmologists for treatments beyond the scope of their legal practice whenever needed [6]. To summarize, optometrists are considered primary eye care professionals while ophthalmologists are secondary level care providers for eye-related conditions.

Educating the patients and turning their attention to the differences between ophthalmologists and optometrists has been identified as an important parameter influencing patient utilization of available eye care services. They both are similar in a certain way where they overlap as well as they both extremely vary in the services offered by these providers [2,7–10]. The lack of knowledge among the ophthalmic patients might overlap the functions among these practitioners leading to poor outcomes. Suffice information about eye care providers could minimize the cost and save plenty of time. In advance, it can reduce the eye morbidity from any possible delays. Fully educated and knowledgeable patients, that we are aiming to have as an outcome to this research, know well enough as to which provider to see for their eye-related condition may minimize morbidity from delays in care and can mitigate issues related to access to care [11–16].

Prior studies suggest that the knowledge toward the differences between ophthalmologists and optometrists is lacking [10, 11, 17, 18]. To our knowledge, there is limited contemporary literature about the public's awareness in regard to the differences between ophthalmologists and optometrists in Saudi Arabia. This study aimed at assessing the public's knowledge of the differences between ophthalmologists and optometrists among the residents of Riyadh, Saudi Arabia.

## 2. Materials and Methods

### 2.1. Study Subjects

A randomly selected 2000 Saudis aged between 18 and 60 years were requested to participate in an online-based study. The study was conducted in the Riyadh region, including Riyadh city and its associated counties, Saudi Arabia, from January through February 2018. All participants were selected with the assistance of a marketing organization that accessed the database of the Saudi Telecom Company. Informed consent was obtained from each participant through an online webpage before they filled out the survey questionnaire. The participants were encouraged to fill the questionnaire at their convenience within the study period.

### 2.2. Sample Size

According to the Saudi General Authority for Statistics, the number of Saudi adults living in Riyadh in 2018 is 4,943,447 individuals [19]. The study sample was estimated using the Epi Info calculator [20] developed by the Centers for Disease Control and Prevention (CDC) with an acceptable margin of error equal to 4% and a design effect of 2. The least acceptable sample size was of 1200 responses. The questionnaire was sent to 2000 adults. The responses reached to 1579 responses, which they all have been included in the analysis. After receiving the Institutional Review Board (IRB) ethical approval from King Abdulaziz City for Science and Technology (KACST), surveys were sent to the study population.

### 2.3. Data Collection Tool

An Arabic version of an existing questionnaire was adopted after permission from Eze. et al. [2]. The translation process includes the cross-cultural validation of the questionnaires and then was reviewed by the committee of translation in our institute as well as a senior ophthalmologist and epidemiologist. Typeform, an online survey tool (www.typeform.com), was used to obtain the participants' responses. Recurrent responses from the same participant were prohibited through connecting every response with their reciprocal internet protocol (IP) address in the Typeform webpage.

### 2.4. Statistical Analysis and Questionnaire

#### 2.4.1. Descriptive Statistics

Statistical analysis was performed using R studio v1.2 for Windows. Categorical variables were summarized as counts and percentages, whereas continuous data such as knowledge score and standardized knowledge score were presented as mean ± standard deviation. Demographics were assessed using 6 questions including age, gender, education level, employment status, marital status, possession of health insurance, and living in Riyadh city or its related governance, while clinical information pertaining to the patients (health insurance, using spectacles, and wearing contact lens) were assessed using three questions. The remaining 13 questions assess the knowledge of the participants; seven questions were regarding the specific tasks, with 5 possible answers (ophthalmologist, optometrist, both of them, none, or not sure) and six in the form of statements which concern about the training requirements with three possible answers (yes, no, or not sure).

#### 2.4.2. Knowledge Scores

The primary outcome was participants' knowledge of the difference between ophthalmologists and optometrists. The participant” knowledge status was classified into “knowledgeable” and “not knowledgeable” by using the Likert scale based on the following scoring criteria: knowledge scoring: correct answer = 1; wrong answer = 0. Average knowledge score (standardized knowledge domain score): this was computed using the method defined in the AGREE II INSTRUMENT as [X/Y] × 100%, where *X *=* *obtained score-minimum possible score and *Y *=* *maximum possible score-minimum possible score. Classification of knowledge status: poor knowledge was defined as an average score of less than 50%; fair knowledge when the average score of 50%–<75%; and the good knowledge was considered if the average score of ≥75%. For the purpose of data analysis, scores ≥50% (fair and good knowledge scores) were considered as a satisfactory level of knowledge. Univariate analysis was performed using the chi-square test of association (*X*^2^) to assess whether the knowledge score (dichotomized into 2 groups based on the 50% level) was significantly different from what was expected under the null hypothesis. The participants' characteristics that presented significant associations with knowledge status using univariate analysis were selected and entered into a multivariate logistic regression model to ascertain their independent effect on the knowledge status and to obtain the odds. Two-tailed hypothesis testing was performed, and a significance level of 0.05 was used throughout the analysis.

## 3. Results

Surveys were sent to 2000 people and 1579 responded, for a response rate of 78.9%. Females represented 54% (*n*=753) of the study population. Participants aged 18–25 years represented the majority (43.4%) of the study sample (*n*=686) (Table 1).

**Figure 1:**
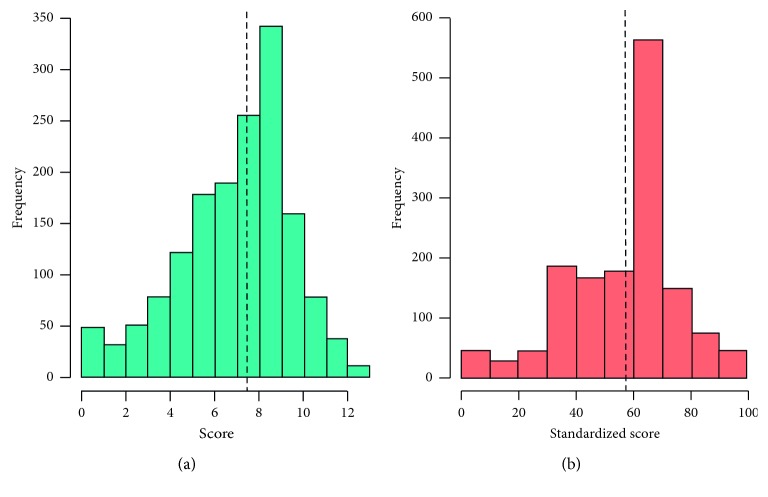
(a) Histogram of unstandardized knowledge score and (b) standardized knowledge score. Lines represent means.

The mean unstandardized knowledge score was 7.42 ± 2.54 standard deviation (SD) while the mean standardized knowledge score was 57.1% ± 19.6%. Both variables were normally distributed as demonstrated by histograms (Figure 1).

Based on the results, almost 50% (*n*=505) of the participants had a fair knowledge (50 to ≤75%) of the differences between ophthalmologists and optometrists while 32% (*n*=505) had poor knowledge (<50%), and only 18% had good knowledge (*n*=285) (Table 2).

The chi-square test results revealed that all variables except having health insurance and gender were significantly associated with having satisfactory knowledge regarding the difference between ophthalmologists and optometrists. Older age was significantly associated with having a better knowledge (*X*^2^=19.952, *p* < 0.001). The odds of having satisfactory knowledge (>50%) in older participants (>26 years) is 1.72 times the odds in younger individuals. The same was noted for higher education (OR=2.026, *X*^2^=40.349, *p* < 0.001) as well as employment status. The odds of having a satisfactory knowledge among participants who were employed or retired was 1.6 times the odds in students or unemployed participants (*X*^2^=17.981, *p* < 0.001). Not wearing contact lenses or spectacles was associated with a lower satisfactory knowledge (OR 0.727 and 0.479, respectively). Finally, not visiting an optometrist in the last 12 months was associated with a lower satisfactory knowledge (OR=0.56, CI: 0.57–0.98) (Table 3).

Only four variables were found to be independent predictors for satisfactory knowledge on multivariate analysis (standardized score >50%): advanced age, higher education, visiting optometrists in the last 12 months, and wearing spectacles (Table 4).

## 4. Discussion

The presence of a significant professional overlap between the ophthalmologist and optometrist creates public misconceptions regarding the specific roles and duties for which each has in the vision health system. To the best of our knowledge, this is the first report addressing public knowledge regarding the ophthalmologists and optometrists' roles in eye care practice among Saudi adults in Riyadh, Saudi Arabia. Previous studies have raised the question, whether the public knowledge is sufficient to know the difference between the ophthalmologist and optometrist in the United States (US) and Nigeria [2, 11, 18, 21, 22]. In the present study, (68%) of the responders were knowledgeable (standardized score >50%) regarding the difference between the ophthalmologist and optometrist. Multivariable logistic regression analysis showed age over 26, higher education level, previous eye examination within 12 months by an optometrist, and wearing spectacles were the only factors that had a significant effect on the level of knowledge among Saudi adults. In Nigeria and the United States, public knowledge of the difference has varied with some studies showing better knowledge than in our cohort and vice versa [2, 10, 23]. In comparison to our study sample, the public knowledge was less in Eze et al. report as it showed 55.6% of the participants were knowledgeable regarding the difference in training requirements and 18.5–74.9% of the participants were knowledgeable about the specific tasks for both ophthalmologist and optometrist in Nigeria [2]. Similarly, Guffay et al. reported 55.6% in the US [10]. However, in the US, Wilson et al. and Bruninga et al. reported 33.0% and 49.0%, respectively [11, 23]. In respect to minor differences in some aspects of the profession that vary from a country to another, as the optometrist in Oklahoma, US, has the advantage to perform some surgical procedures that are not allowed to be done by the optometrist in Saudi Arabia; this study is restricted to the major role and responsibilities of the ophthalmologist and optometrist in Saudi Arabia [24].

Educational level was an important demographic feature that had a major significant effect on satisfactory knowledge level (>50%) as (73.7%) of those with higher education were well rounded of the differences. This was in agreement with previous studies where they illustrated direct association between the level of education and knowledge level [10, 25, 26]. In addition, employment status had a considerable impact on the level of knowledge. Similar findings were observed in other surveys as well [10, 27]. A similar relationship was found with numerous other aspects of health literacy [20].

Those who had previous eye examination had greater knowledge than those who have never been examined, 71.4% and 58.3%, respectively. This finding is self-evident and in agreement with Eze et al. findings [2]. Wearing contact lenses and/or spectacles positively correlate with acceptable knowledge among this study's participants. Wilson et al. reported similar observations [11]. On the contrary, Eze et al. reported that the level of satisfactory knowledge was higher among those who did not wear contact lens and spectacles [2]; this finding could be explained by using of ready-made spectacles without prior standard refractive eye examination and uncommon use of contact lens due to the dusty and unstable weather in the study area. Respondents who are over the age of 26 years were more knowledgeable than those younger than 26, that is likely due to generally increased knowledge and a higher probability of having had an eye exam as part of getting older. Additionally, married participants were knowledgeable (73.2%) more than singles (63.5%). This is could be explained by higher levels of awareness and attitude regarding eye diseases among Saudi adults [28].

## 5. Conclusions

This study showed a relatively low level of public knowledge of the difference between the role of the ophthalmologist and optometrist among a Saudi population. There is an essential demand from the policy maker and ministry of health for awareness enhancement through campaigns and social media regarding eye care provider's duties and practices.

## Figures and Tables

**Table 1:** Age and sex distribution of the adult respondents of Riyadh, Saudi Arabia, in 2018 (*n*=1579).

	Male		Female		Total	
Age	*n*	%	*n*	%	*n*	%
18–25	321	46.8	365	53.2	686	43.4
26–35	202	53.7	174	46.3	376	23.8
>36	203	39.3	314	60.7	517	32.7
Total	726	46	853	54		

**Table 2:** Average (standardized) knowledge score categories of the adult respondents of Riyadh, Saudi Arabia, in 2018 (*n*=1579).

Variable	*n*	%
Poor knowledge (<50%)	505	32
Fair knowledge (50 to ≤75%)	789	50
Good knowledge (≥75%)	285	18

**Table 3:** Factors associated with participants' satisfactory knowledgic knowledge about the difference between ophthalmologists and optometrists of the adult respondents of Riyadh, Saudi Arabia in 2018 (*n*=1579) (univariate analysis).

Variable	Value	Satisfactory knowledge >50%		*X* ^2^	*p*	OR	Lower 95% CI	Upper 95% CI
		*n*	%					
Gender	Male	227	31.3	0.26	0.612	0.941	0.761	1.164
	Female	278	32.6					
Age	>26	126	24.4	19.95	<0.001^*∗*^	1.722	1.359	2.182
	<26	379	35.7					
Education	Higher education	266	26.3	40.35	<0.001^*∗*^	2.026	1.630	2.518
	Lower education	239	42.0					
Employment	Employed/retired	190	26.5	17.98	<0.001^*∗*^	1.603	1.292	1.990
	Student/unemployed	315	36.6					
Health insurance	No	351	32.50	0.349	0.555	0.927	0.738	1.165
	Yes	154	30.86					
Marital status	Married	198	26.79	16.751	<0.001^*∗*^	1.574	1.269	1.951
	Single	307	36.55					
Wearing contact lens	No	346	34.46	7.484	<0.05^*∗*^	0.727	0.581	0.910
	Yes	159	27.65					
Wearing spectacles	No	259	41.84	44.753	<0.001^*∗*^	0.479	0.386	0.594
	Yes	246	25.62					
Visited optometrist	No	169	41.73	23.186	<0.001^*∗*^	0.560	0.443	0.708
	Yes	336	28.62					

OR: odds ratio; ^*∗*^statistically significant.

**Table 4:** Independent predictors of participants' satisfactory knowledge in the public knowledge toward the differences between ophthalmologists and optometrists among the adult respondents of Riyadh, Saudi Arabia, in 2018 (*n*=1579) (multivariate analysis).

Variable	Value	OR	95% CI	B	SE	*p*
Age	>26	1.73	(1.17–1.19)	0.4	0.125	<0.001^*∗*^
Education	Higher education	1.92	(1.54–2.4)	0.653	0.114	<0.001^*∗*^
Visited optometric	No	0.75	(0.57–0.98)	−0.287	0.137	<0.05^*∗*^
Wearing spectacles	No	0.57	(0.45–0.73)	−0.563	0.125	<0.001^*∗*^

^*∗*^Statistically significant.

## Data Availability

The data used to support the findings of this study are available from the corresponding author upon request.
